# Dental Students’ Knowledge, Confidence, Ability, and Self-Reported Difficulties in Periodontal Education: A Mixed Method Pilot Study

**DOI:** 10.3390/dj10040063

**Published:** 2022-04-06

**Authors:** Amirsalar Mofidi, Arnaldo Perez, Ida Kornerup, Liran Levin, Silvia Ortiz, Hollis Lai, Jacqueline Green, Seongju Kim, Monica P. Gibson

**Affiliations:** Faculty of Medicine and Dentistry, School of Dentistry, University of Alberta, Edmonton, AB T6G 1C9, Canada; mofidi@ualberta.ca (A.M.); perezgar@ualberta.ca (A.P.); kornerup@ualberta.ca (I.K.); liran@ualberta.ca (L.L.); ssortiz@ualberta.ca (S.O.); hollis.lai@ualberta.ca (H.L.); jgreen@ualberta.ca (J.G.); seongju@ualberta.ca (S.K.)

**Keywords:** dental education, methodological study, survey methodology, periodontal diseases, periodontitis, periodontal index, periodontics, pilot projects, qualitative research

## Abstract

Evidence on periodontal education areas in which students have difficulties and their factors are limited. In this study, third- and fourth-year dental students’ knowledge was assessed as well as their confidence and ability in five periodontal educational areas using a mixed-method approach. A survey was used to collect data related to history-taking, medical examination, diagnosis, treatment planning, and follow-up. Student answers were compared to the consensual answers of an expert panel using the cosine-similarity index (CSI). Descriptive statistics assessed confidence and ability for diagnosis. Semi-structured individual interviews were used to collect data on reported reasons for difficulties in periodontal education. A content analysis was employed to analyze the interview data. Eighteen third- and fourth-year dental students completed the survey and eleven were interviewed. Students’ knowledge was adequate regarding diagnosis and treatment planning. Third-year students’ median CSI were 0.93 and 0.89, respectively. Fourth-year students’ median CSI were 0.9 and 0.93, respectively. Students felt confident in history-taking and examination but lacked confidence and ability in diagnosis and treatment planning. Reported reasons for difficulties in periodontal education were linked to both preclinical and clinical pedagogical issues. Further improvements in preclinical and clinical periodontal education are needed to address students’ lack of knowledge, confidence, and skills in key periodontal areas.

## 1. Introduction

Periodontal diseases are highly prevalent worldwide, ref. [1] affecting almost half of those aged 30 and older and 70% of those aged 65 and older [2]. People with periodontal disease are at a higher risk of experiencing many adverse consequences such as tooth loss, pain, and low self-esteem [3,4,5]. Periodontal diseases have also been associated with systemic conditions, such as diabetes and cardiovascular diseases [6,7].

Periodontal diseases are manageable with a timely diagnosis, proper treatment, and maintenance plans. However, research has found that general dentists may misdiagnose periodontal diseases and fail to make timely referrals for specialized care, thus limiting the effectiveness of interventions [8,9,10,11]. A decreased confidence in managing advanced periodontal diseases among general dentists has also been reported [12].

As future general dentists, students share those expectations upon completing their training. To date, there is scant literature on students’ readiness for periodontal care and their difficulties with periodontal diagnosis and management [13,14,15]. Specifically, research has found that students have greater difficulties with diagnosis over management, and their abilities to diagnose and formulate treatment plans improve over time [15,16]. The importance of early diagnoses is emphasized in early intervention using different approaches, such as localized antibiotics delivery and laser therapies.

Further research is needed to identify the specific areas in which students have difficulties. For example, correct diagnosis depends on well-conducted history-taking and examination, adequate interpretation of information, and specific knowledge about disease identities, including definitions, diagnostic criteria, treatment options, and risk factors [17,18]. Assessing knowledge, confidence, attitude, and skill within specific areas is also important to define the targets of interventions aimed at improving periodontal education. Few studies have investigated these issues, especially in periodontal treatment planning and referral-making, highlighting the need for further evidence [13,16].

Student views of the challenges they experience throughout their periodontal training can help to define the targets of remedial interventions in periodontal education. A recent study on students’ self-assessments of their periodontal care demonstrates the value of student perceptions for identifying student-, faculty-, and school-related factors that may account for their suboptimal performance [19]. In addition to increasing the understanding of educational issues, the use of student perspectives has been successful in improving curriculum content, delivery, and assessment in higher education [20]. The purpose of the study was to identify strengths and weaknesses in didactic and clinical periodontal training in third- and fourth-year dental students. Therefore, the objectives of the study were to determine students’ knowledge, confidence, and abilities related to periodontal diagnosis and treatment planning so as to explore their views of factors accounting for their difficulties in periodontal education. The combined quantitative and qualitative data will help unearth specific areas for improving periodontal education, which has similar characteristics across dental schools.

## 2. Materials and Methods

### 2.1. Design

This study was guided by the sequential explanatory mixed-methods design. Quantitative assessment was used to identify areas of interest, followed by a qualitative assessment to deeply investigate the problems [21]. Ethics approval for the study was obtained from the University of Alberta Research Board (Pro00071317).

### 2.2. Participants

Participants were third- (Y3) and fourth-year (Y4) dental students during the 2017/2018 school year at the School of Dentistry, University of Alberta. Their periodontal training begins in the second year and includes didactic lectures, a simulation laboratory, and clinical rotations. Students were invited to participate by the delivery of emails and classroom presentations. The recruitment was conducted by the study coordinator (J.G.), who was a neutral party not responsible for grading the students. The coordinator had extensive training in qualitative studies.

### 2.3. Data Collection

Informed consent was obtained prior to data collection, which occurred after the students completed didactic and clinical training. To determine areas of difficulties in periodontal diagnosis and treatment planning, students’ knowledge and confidence were examined in five key areas of clinical performance in periodontal care [22,23], namely history-taking, examination, diagnosis, treatment, and follow-up. These areas are vital parameters of clinical performance and are competencies dental students should possess upon graduation [24]. Additionally, students’ abilities to formulate a correct periodontal diagnosis and treatment planning were assessed.

Students were asked to complete an online survey through Research Electronic Data Capture (REDCap software, Vanderbilt University, Nashville, TN, USA) [25] in a classroom setting. The development of the survey was informed by the literature on periodontal dental education and consisted of eight questions, including three clinical cases [14,26]. Questions 1 to 5 assessed confidence and knowledge regarding the periodontal areas. Here, students were asked to rate their level of confidence in each area using a five-point scale and to elaborate. Questions 5 to 8 presented students with three clinical cases, which included medical and dental history, chief complaints, clinical photos, radiographs, and charting. In these questions, students were asked to diagnose each case without predefined options (e.g., what is your best diagnosis with the information provided?) and to formulate a treatment plan for each. Lane et al. [14] designed the study to investigate periodontal diseases commonly seen in general practice. They used expert panel (*n* = 10) consensus to develop the correct responses. Armitage’s 1999 diagnostic classification system was used. This study was conducted prior to the release of the new classification proposed by the AAP/EOP world workshop [26].

Semi-structured individual interviews were used to collect data on reasons for clinical difficulties using a conversational format. A pilot-tested interview guide was developed by considering general recommendations for interviewing and quantitative findings. The interviews lasted approximately 30 min and were held in a private room at the school clinic. 

### 2.4. Data Analysis

Quantitative analysis was performed with SPSS 24 [27]. Bonferroni correction was employed to minimize type 1 error and Bootstrap was used to correct for non-normal distribution and the small sample size.

Cosine-Similarity Index (CSI) was used to assess the similarity between students’ answers and the gold standard (experts’ answers). Here, the student response text is first represented by vectors and then compared to the gold standard vector using the resulting cosine measure [28]. The closer student responses are to 1, the greater similarity to the gold standard for that question. Correctness of student answers was set at 65%—the passing grade for clinical courses at the school. Descriptive statistics were used to calculate the percentages of correct diagnoses. Three levels of student confidence were defined: not confident (>3), somewhat confident (2–3), and confident (<2). 

The Independent-Sample Median Test was used to compare median differences between student years in knowledge and ability to formulate treatment planning. The significance level was set at Bonferroni corrected (*p* = 0.01). Pearson Chi-square statistical test was used to compare the two groups regarding their ability to diagnose the clinical cases.

Interviews were audio-recorded and transcribed verbatim by Transcript Heroes© (https://transcriptheroes.ca/, accessed on 15 September 2019). Interview data were analyzed using content analysis [29] with NVivo 12 [30]. After familiarization with the transcript data, codes were assigned to text segments. The assigned codes were then grouped into categories and subcategories of difficulties in a cyclical manner. Representative quotes were selected to illustrate the identified student difficulties. Two researchers (AM and AP) conducted the analysis of the qualitative data, which was later discussed with the entire research team. Discrepancies regarding data analysis were solved by consensus.

## 3. Results

Eighteen Y4 and 34 Y3 dental students completed the anonymous survey for an overall response rate of 66.67%. Table 1 shows that the median of Y3 students successfully answered the knowledge questions related to diagnosis (0.93, Confidence Interval [CI] = 0.93–0.93) and treatment (0.89, CI = 0.62–0.89), but not medical history (0.57, CI = 0.52–0.65), examination (0.54, CI = 0.44–0.57), and follow-up (0.27, CI = 0.08–0.32). Similarly, the median of Y4 students correctly answered the questions related to diagnosis (0.90, CI = 0.79–0.90) and treatment (0.94, CI = 0.93–0.93), while their answers to examination (0.67, CI = 0.62–0.70) and medical history questions (0.65, CI = 0.61–0.71) were just acceptable. Conversely, knowledge about follow-up (0.46, 0.00–0.50) was suboptimal. Statistically significant (*p* < 0.001) differences were observed between Y3 and Y4 students regarding knowledge about examination, diagnosis, and treatment.

The median Y3 students felt somewhat confident in all areas. Similarly, Y4 felt somewhat confident in diagnosis (M = 2.0), treatment (M = 2.0) and follow-up (M = 2.0), but confident in history-taking (M = 1.5) and examination (M = 1.0). No statistically significant differences were observed between Y3 and Y4 students regarding confidence (Table 1).

As depicted in Table 2, 79.4% of Y3 students correctly diagnosed generalized severe chronic periodontitis but failed to diagnose gingivitis (47.1%) and localized aggressive periodontitis (44.1%). Conversely, 50% of Y4 students correctly diagnosed gingivitis and localized aggressive periodontitis, while 66.7% correctly diagnosed generalized severe chronic periodontitis. No statistically significant differences in diagnosing the three periodontal cases were observed between both years. In questions about managing gingivitis, generalized severe chronic periodontitis and localized aggressive periodontitis, the median of all students was found to be below the acceptable level.

Eleven students were interviewed, two of whom were female and three were male Y3 students ranging in age from 23 to 27 years, and three female and male Y4 students ranging in age from 24 to 28 years. Table 3 displays representative quotes supporting reported reasons for difficulties regarding periodontal performance.

### 3.1. Preclinical Education Reasons

Students highlighted the insufficient coverage of relevant content in preclinical training as a reason for struggling to provide proper periodontal care to patients. Students felt unprepared in several areas, including risk management, diagnosis, and treatment options. They felt unfamiliar with treatment options for patients unresponsive to initial therapy and unprepared to determine when a referral to a periodontist was necessary. As future general dentists, students stated that they do not require knowledge on performing surgical procedures but should be aware of surgical options for referrals to specialized care and to educate patients regarding available treatment options.

Students mentioned that the relevant content is not always properly delivered, contributing to decreased performance in periodontics. For instance, discussing complex concepts before basic ones. This negatively influences student learning, interest in periodontics, and the perceived importance of the topics covered. 

Students’ difficulties were also attributed to insufficient practice time allotted to periodontal procedures in simulation labs when compared to other specialties. This narrowed student opportunities to learn from instructor feedback when developing psychomotor clinical skills.

### 3.2. Clinical Education Reasons

Several students mentioned instructor inconsistencies as a reason for suboptimal performance because inconsistencies between instructors regarding diagnosis, risk assessments, treatment, and maintenance undermine student understanding of proper periodontal care. Next, students stated that issues with assessing treatment outcomes are attributed to patient attrition and the time lag between care delivery and re-evaluation. Lastly, the assignment of patients with complex treatment needs at the beginning of student clinical tenure makes students feel unable to provide proper periodontal care and to manage complex cases.

## 4. Discussion

The study found that students had limited knowledge in less complex areas (e.g., examination) and lacked confidence and ability in more complex areas (e.g., diagnosis). The reported reasons for these difficulties were attributed to preclinical and clinical periodontal education issues and not to student characteristics. In Canada, students undergo 4 years of dental school. Periodontics is taught in all years, with a focus on didactic learning in the first 3 years and clinical practice during years 3 and 4. Previous studies focused on student performance in diagnosis and treatment, yielding insufficient data on students’ knowledge and confidence, thus failing to comprehensively explore student views on challenges in periodontal education [14,15]. Early intervention with proper training and knowledge are key factors in the successful treatment planning and the prognosis of periodontal diseases.

The findings suggest that students require continual didactic and clinical periodontal education to reinforce basic concepts and improve performance. These data, however, must be interpreted with caution, as students may be able to perform the activities in which they claim to have insufficient knowledge. Miller’s model of assessing clinical competencies suggests that knowledge about an activity may not predict performance and vice versa [31]. Student knowledge may be influenced by several factors, such as the amount of information received, time lag between knowledge delivery and assessment, and emphasis on some knowledge areas. Indeed, students who participated in the study received more information on diagnosis and treatment planning than other areas, which may explain the observed level of knowledge in each.

Furthermore, Y4 students appeared to have more formal knowledge than Y3 students in most of the periodontal areas examined, as consistent with previous reports [14,16]. However, Y3 students were more knowledgeable about diagnosis than Y4 students, which may be due to a recency effect, as Y3 students reviewed and discussed the classification system for periodontal diseases closer to the time of data collection than their Y4 counterparts.

Students had limited knowledge and confidence regarding follow-up. Follow-up is important for chronic-disease treatment as regular visits with care providers lead to the effective management of health [32]. Providing students with the necessary knowledge and skills in this area supports them in the role they are expected to play in addressing the increased burden of chronic oral health diseases [33]. Moreover, the data highlight the need to improve students’ recognition of periodontal risk factors, as previously reported [16]. 

Students’ decreased confidence and ability to diagnose and manage common periodontal conditions is associated with motivation, behavior, and performance [34]. Improving confidence in periodontal diagnosis and treatment can be achieved simultaneously with critical skill-building through active learning, which has proven effective in improving self-efficacy and performance [35].

Both student groups incorrectly diagnosed the aggressive periodontitis case. This condition has a particular set of characteristics (e.g., early onset, rapid attachment loss), which makes its presentation unique [26]. Similarly, Lane et al. [14] found that only 49% of Y3 students and 56% of Y4 students correctly diagnose aggressive periodontitis. Future periodontal courses should prepare students for diagnosing and managing aggressive periodontitis. Case-based learning using authentic cases [36] and other active learning strategies [37] can help enhance students’ understanding, recognition, and management of the periodontal disease. 

The findings regarding students’ ability to properly diagnose and formulate treatment plans differ from previous research [14]. This inconsistency may be due to asking students to provide, rather than select, a diagnosis and treatment plan for each case, allowing for an improved assessment of students’ abilities in these areas.

Challenges students experience in periodontal education are likely influenced by an interplay of factors at individual, interpersonal, course, program, and organizational levels. Preclinical and clinical factors that students uncovered complement those that Chandrasekaran et al. [19] identified in their study, where students identified several factors, including the involvement of multiple providers in patient care, emphasis on academic requirements, limited clinical experiences, preference for other areas, and poor patient compliance.

Previous research has also identified students’ insufficient preparation for clinical rotations [38] and a lack of instructor calibration as negative influences on student clinical performance. Evidence-based guidelines, consensus training programs, and calibration meetings have been suggested to address these issues [14,39]. Collectively, the strength of the study was the robust methodology of using both qualitative and quantitative assessments in addressing those issues.

The limitations that students identified in periodontal education are similar to those of traditional dental curricula, including content fragmentation, irrelevant content, lecture-based teaching, insufficient clinical experiences, and inadequate assessment of clinical competencies [40,41,42]. To address these issues, the Commission on Dental Accreditation made several recommendations with the intention to improve dental education [43]. These include adopting a competency-based approach, active learning, vertical and horizontal curricular integration, standardizing assessment methods, calibrating faculty, and exposing students to clinical experiences earlier in their programs. However, the implementation of these recommendations remains challenging in dental education where resistance to educational innovations is common. 

The first study limitation is that although only one question assessed student knowledge in each area, the questions focused on student approach. Questions were open-ended, allowing for sufficient detail in answers. Second, there was a small sample of Y4 students, which is expected due to the workload of senior students. Third, although common in education research, featuring a single institution limits the generalizability of findings [44]. Fourth, while only three cases were used to assess students’ abilities to diagnose and formulate treatment plans, these were obtained from the available literature and represented common periodontal conditions with different levels of severity. Finally, this study was conducted before the new classification of periodontal diseases was completely adapted, causing the students to encounter difficulties in identifying aggressive periodontitis cases. As a future direction, a similar study should be conducted using the current diagnostic classification system.

## 5. Conclusions

Although students’ knowledge was acceptable regarding diagnosis and treatment planning, further improvements in periodontal education are needed to address deficiencies in essential areas. Similarly, a combination of proper didactic teaching, clinical exposure, and skill development can improve students’ perceived and actual abilities in periodontics. Evidence-based recommendations made by dental education organizations can be used to address the preclinical and clinical issues that students raised in the study. 

## Figures and Tables

**Table 1:** Knowledge and confidence in the five periodontal areas.

	Knowledge			Confidence		
Areas	Y3 Median CSI, CI	Y4 Median CSI, CI	*p*-Value *	Y3 Median, CI	Y4 Median, CI	*p*-Value *
Medical History	0.57, (0.52, 0.65)	0.65, (0.61, 0.70)	0.041	2.00, (1.00, 2.00)	1.50, (1.00, 2.00)	0.224
Periodontal examination	0.54, (0.44, 0.57)	0.67, (0.62, 0.70)	0.001	2.00, (2.00, 2.00)	1.00, (1.00, 2.00)	0.224
Diagnosis	0.93, (0.93, 0.93)	0.90, (0.79, 0.90)	0.001	2.00, (2.00, 2.00)	2.00, (1.50,2.00)	0.100
Treatment	0.89, (0.62, 0.89)	0.93, (0.93, 0.93)	0.001	2.00, (2.00, 2.00)	2.00, (2.00, 2.00)	0.820
Follow-up	0.27, (0.08, 0.32)	0.46, (0.00, 0.50)	0.200	2.00, (2.00, 3.00)	2.00, (2.00, 2.00)	0.289

* *p*-values based on Independent-Samples Median Test. Cosine-Similarity Index (CSI). Confidence Interval (CI).

**Table 2:** Diagnosis and treatment planning of the three clinical cases.

	Diagnosis			Treatment Planning		
Cases	Y3 (%)	Y4 (%)	*p*-Value **	Y3 Median CSI, CI	Y4 Median CSI, CI	*p*-Value *
Gingivitis	47.1	50.0	0.840	0.53, (0.51, 0.54)	0.36, (0.34, 0. 44)	0.009
Generalized Severe Chronic Periodontitis	79.4	66.7	0.313	0.40, (0.33, 0.45)	0.61, (0.03, 0.61)	0.001
Localized Aggressive Periodontitis	44.1	50.0	0.686	0.36, (0.12, 0.43)	0.16, (0.08, 0.31)	0.382

** *p*-values based on Pearson Chi Square. * *p*-values based on Independent-Samples Median Test.

**Table 3:** Reasons for difficulties in periodontal education.

Categories	Reasons	Representative Quotes
Preclinical reasons	Insufficient coverage of relevant content	“I encourage them to quit smoking and explain the risk factors and how it’s making their periodontal disease worse, but we don’t really have any resources here at the school for them, and we never really get taught how to do smoking cessation with patients” (Y3 Student 4)
	Inadequate delivery of relevant content	“Before we even know what attached gingiva is, before any of like that, we’re talking about like these advanced like studies into like chronic perio and like the bacterial subtypes” (Y4 Student 2)
	Insufficient simulation of clinical skills	“So like in any part of dentistry, we’ve had practical competencies in operative, fixed, pretty much even in dentures, and then we would finish the competency in SimLab, and now we are allowed to treat in clinic. That didn’t happen in perio, so perio didn’t actually give us that option, someone to give us feedback on” (Y4 Student 5)
Clinical reasons	Instructor inconsistency	“Periodontists on staff, a lot of them see it different ways. So, you get some individuals that think it’s moderate, some individuals that might think it might be aggressive at one region, and some people that think that this might be just a varying form of gingivitis” (Y4 Student 3)
	Inability to assess treatment outcomes	“I don’t know if that defeats the purpose of the re-evaluation, but I find myself doing the re-evaluation and sometimes going back to initial therapy, and I don’t know if that’s because initial therapy didn’t work or if that was because we didn’t have the re-evaluation soon enough” (Y3 Student 1)
	Mismatch between complexity of the case and student clinical experience	“And so then we get to clinic and see these high-risk cases: One, we’re like not prepared to treat them; we don’t have that much experience because our technique is terrible” (Y3 Student 3)

## Data Availability

The data presented in this study are available on request from the corresponding author. The data are not publicly available due to privacy restrictions.

## References

[1] FDI World Dental Federation (2015). The Challenge of Oral Disease—A Call for Global Action. The Oral Health Atlas.

[2] Eke P.I., Dye B.A., Wei L., Thornton-Evans G.O., Genco R.J. (2012). Prevalence of periodontitis in adults in the United States: 2009 and 2010. J. Dent. Res..

[3] Sanz M., Ceriello A., Buysschaert M., Chapple I., Demmer R.T., Graziani F., Herrera D., Jepsen S., Lione L., Madianos P. (2018). Scientific evidence on the links between periodontal diseases and diabetes: Consensus report and guidelines of the joint workshop on periodontal diseases and diabetes by the International Diabetes Federation and the European Federation of Periodontology. Diabetes Res. Clin. Pract..

[4] Buset S.L., Walter C., Friedmann A., Weiger R., Borgnakke W.S., Zitzmann N.U. (2016). Are periodontal diseases really silent? A systematic review of their effect on quality of life. J. Clin. Periodontol..

[5] Herrera D., Retamal-Valdes B., Alonso B., Feres M. (2018). Acute periodontal lesions (periodontal abscesses and necrotizing periodontal diseases) and endo-periodontal lesions. J. Clin. Periodontol..

[6] Al-Harthi L., Cullinan M., Leichter J., Thomson W. (2013). The impact of periodontitis on oral health-related quality of life: A review of the evidence from observational studies. Aust. Dent. J..

[7] Tonetti M.S., Eickholz P., Loos B.G., Papapanou P., Van Der Velden U., Armitage G., Bouchard P., Deinzer R., Dietrich T., Hughes F. (2015). Principles in prevention of periodontal diseases: Consensus report of group 1 of the 11th European Workshop on Periodontology on effective prevention of periodontal and peri-implant diseases. J. Clin. Periodontol..

[8] McGuire M.K., Scheyer E.T. (2003). A referral-based periodontal practice—Yesterday, today, and tomorrow. J. Periodontol..

[9] Lee J.H., Bennett D.E., Richards P.S., Inglehart M.R. (2009). Periodontal referral patterns of general dentists: Lessons for dental education. J. Periodontol..

[10] Dockter K.M., Williams K.B., Bray K.S., Cobb C.M. (2006). Relationship between prereferral periodontal care and periodontal status at time of referral. J. Periodontol..

[11] Cobb C.M., Carrara A., El-Annan E., Youngblood L.A., Becker B.E., Becker W., Oxford G.E., Williams K.B. (2003). Periodontal referral patterns, 1980 versus 2000: A preliminary study. J. Periodontol..

[12] Darby I., Angkasa F., Duong C., Ho D., Legudi S., Pham K., Welsh A. (2005). Factors influencing the diagnosis and treatment of periodontal disease by dental practitioners in Victoria. Aust. Dent. J..

[13] Williams K.B., Burgardt G.J., Rapley J.W., Bray K.K., Cobb C.M. (2014). Referring periodontal patients: Clinical decision making by dental and dental hygiene students. J. Dent. Educ..

[14] Lane B.A., Luepke P., Chaves E., Maupome G., Eckert G.J., Blanchard S., John V. (2015). Assessment of the calibration of periodontal diagnosis and treatment planning among dental students at three dental schools. J. Dent. Educ..

[15] Friesen L.R., Walker M.P., Kisling R.E., Liu Y., Williams K.B. (2014). Knowledge of risk factors and the periodontal disease-systemic link in dental students’ clinical decisions. J. Dent. Educ..

[16] John V., Lee S.J., Prakasam S., Eckert G.J., Maupome G. (2013). Consensus training: An effective tool to minimize variations in periodontal diagnosis and treatment planning among dental faculty and students. J. Dent. Educ..

[17] Bader J.D., Shugars D.A. (1992). Understanding dentists’ restorative treatment decisions. J. Public Health Dent..

[18] Northridge M.E., Kumar A., Kaur R. (2020). Disparities in access to oral health care. Annu. Rev. Public Health.

[19] Chandrasekaran S., Powell C., De la Rosa L., Mittal A., Johnson L. (2017). Dental students’ reflections on quality of periodontal care in dental school clinics. J. Dent. Ed..

[20] Yao Y., Grady M.L. (2005). How do faculty make formative use of student evaluation feedback?: A multiple case study. J. Person. Eval. Educ..

[21] Creswel J. (2009). Research Design: Qualitative, Quantitative, and Mixed Methods Approaches.

[22] Sweeting L.A., Davis K., Cobb C.M. (2008). Periodontal treatment protocol (PTP) for the general dental practice. J. Am. Dent. Hyg. Assoc..

[23] Pihlstrom B.L. (2001). Periodontal risk assessment, diagnosis and treatment planning. Periodontol. 2000.

[24] Sanz M., Meyle J. (2010). Scope, competences, learning outcomes and methods of periodontal education within the undergraduate dental curriculum: A consensus report of the 1st European workshop on periodontal education–position paper 2 and consensus view 2. Eur. J. Dent. Educ..

[25] Harris P.A., Taylor R., Thielke R., Payne J., Gonzalez N., Conde J.G. (2009). Research electronic data capture (REDCap)—A metadata-driven methodology and workflow process for providing translational research informatics support. J. Biomed. Inform..

[26] Tonetti M.S., Greenwell H., Kornman K.S. (2018). Staging and grading of periodontitis: Framework and proposal of a new classification and case definition. J. Periodontol..

[27] IBM Corp (2016). IBM SPSS Statistics for Windows, Version 24.0.

[28] Singhal A. (2001). Modern information retrieval: A brief overview. IEEE Database Eng. Bull..

[29] Elo S., Kyngäs H. (2008). The qualitative content analysis process. J. Adv. Nurs..

[30] QSR International Pty Ltd. (2018). NVivo (Version 12). https://www.qsrinternational.com/nvivo-qualitative-data-analysis-software/home.

[31] Miller G.E. (1990). The assessment of clinical skills/competence/performance. Acad. Med..

[32] Sanz M., Bäumer A., Buduneli N., Dommisch H., Farina R., Kononen E., Linden G., Meyle J., Preshaw P.M., Quirynen M. (2015). Effect of professional mechanical plaque removal on secondary prevention of periodontitis and the complications of gingival and periodontal preventive measures: Consensus report of group 4 of the 11th European Workshop on Periodontology on effective prevention of periodontal and peri-implant diseases. J. Clin. Periodontol..

[33] Jin L., Lamster I., Greenspan J., Pitts N., Scully C., Warnakulasuriya S. (2016). Global burden of oral diseases: Emerging concepts, management and interplay with systemic health. Oral Dis..

[34] Trafimow D., Sheeran P., Conner M., Finlay K.A. (2002). Evidence that perceived behavioural control is a multidimensional construct: Perceived control and perceived difficulty. Br. J. Soc. Psychol..

[35] Wu F., Sheng Y. (2019). Social support network, social support, self-efficacy, health-promoting behavior and healthy aging among older adults: A pathway analysis. Arch. Gerontol. Geriatr..

[36] Hunt T., Jones T.A., Carney P.A. (2020). Peer-Assisted Learning in Dental Students’ Patient Case Evaluations: An Assessment of Reciprocal Learning. J. Dent. Educ..

[37] Ganatra S., Doblanko T., Rasmussen K., Green J., Kebbe M., Amin M., Perez A. (2020). Perceived Effectiveness and Applicability of Think-Pair-Share Including Storytelling (TPS-S) to Enhance Clinical Learning. Teach. Learn. Med..

[38] Gilmour A., Welply A., Cowpe J., Bullock A.D., Jones R.J. (2016). The undergraduate preparation of dentists: Confidence levels of final year dental students at the School of Dentistry in Cardiff. Br. Dent. J..

[39] Lanning S.K., Pelok S.D., Williams B.C., Richards P.S., Sarment D.P., Oh T.J., McCauley L.K. (2005). Variation in periodontal diagnosis and treatment planning among clinical instructors. J. Dent. Educ..

[40] Kassebaum D.K., Hendricson W.D., Taft T., Haden N.K. (2004). The dental curriculum at North American dental institutions in 2002–03: A survey of current structure, recent innovations, and planned changes. J. Dent. Educ..

[41] DePaola D.P., Slavkin H.C. (2004). Reforming dental health professions education: A white paper. J. Dent. Educ..

[42] Lee J.S., Somerman M.J. (2018). The Importance of Oral Health in Comprehensive Health Care. JAMA.

[43] Commission on Dental Education (2006). Accreditation Standards for Dental Education Programs.

[44] Carney P.A., Brandt B., Dekhtyar M., Holmboe E.S. (2018). Advancing health professions education research by creating a network of networks. Acad. Med..

